# A Novel Diagnostic Tool for West Nile Virus Lineage 1a and 2 Using a CRISPR-Cas12a System

**DOI:** 10.3390/bios15120807

**Published:** 2025-12-10

**Authors:** Soo Bin Hwang, Yoon-Jae Song, Pil-Gu Park

**Affiliations:** Department of Life Science, Gachon University, 1342 Seongnam-Daero, Seongnam-Si 13120, Gyeonggi-Do, Republic of Korea; subinghkd@naver.com (S.B.H.); songyj@gachon.ac.kr (Y.-J.S.)

**Keywords:** West Nile virus, diagnosis, CRISPR-Cas12a, DETECTR

## Abstract

The West Nile Virus (WNV), transmitted by Culex mosquitoes as a major vector, has been reported worldwide. Also, West Nile neuroinvasive disease (WNND) caused by WNV lineage 1a and 2 neuroinvasive infections has been constantly reported with high fatality rates. Nevertheless, there are no treatments and vaccinations, so diagnosis in the early stages is important. Recently, a molecular diagnostic technique using DNA endonuclease-targeted CRISPR trans reporter (DETECTR) with the CRISPR-Cas12a system integrated with isothermal nucleic acid amplification has newly emerged. In this study, we designed a 2-Step WNV DETECTR with reverse transcription–recombinase polymerase amplification (RT-RPA) for rapid and sensitive WNV diagnosis. It successfully detected down to 1.0 × 10^2^ RNA copies for both WNV lineage 1a and 2 with demonstrating similar sensitivity to qRT-PCR without cross-reactivity to other viruses. Additionally, we designed a 1-Step WNV DETECTR, incorporating all processing steps into a single tube, capable of detecting down to 1.0 × 10^3^ RNA copies for both lineages. Furthermore, we developed a more streamlined method, the 1-Step with Filter WNV DETECTR, which achieved detection limits comparable to the 2-Step method, while reducing the processing time by 5 min. This study also explored the potential of the Punch-it™ NA-Sample Kit as an efficient alternative lysis method by comparing the detection differences across various lysis methods. Through this method, we achieved rapid and simple amplification and detection processes suitable for field diagnostics with high specificity and sufficient sensitivity. Therefore, DETECTR methods presented themselves as promising alternatives to conventional diagnostic tools, potentially overcoming financial and technical constraints in diverse medical settings.

## 1. Introduction

West Nile Virus (WNV) is an arbovirus (arthropod-borne virus) first isolated in 1937 from an adult in the West Nile district of Uganda and has been reported globally with significant incidences across continents [1,2,3]. Its widespread distribution is largely explained by the ecology of its transmission cycle, in which birds serve as the primary hosts and Culex mosquitoes act as vectors [4], while humans and other vertebrates are typically infected as dead-end hosts, with a few reports of possible human-to-human transmission [5,6,7,8,9]. In humans, WNV infection ranges from asymptomatic infection—accounting for roughly 80% of cases—to West Nile fever (WNF), a systemic febrile illness, and in fewer than 1% of infections, to West Nile neuroinvasive disease (WNND) [10], which includes meningitis (West Nile meningitis; WNM), encephalitis (West Nile encephalitis; WNE), and poliomyelitis-like syndromes (West Nile poliomyelitis; WNP) [11,12], more frequently in older or immunocompromised individuals and often associated with considerable mortality during outbreaks [13]. Despite this clinical issue, definitive vaccines or treatments for WNV have been absent, and viremia in infected persons is typically transient and may have declined by the time neurological symptoms appear [10]. As a result, it underscores the importance of sensitive and rapid diagnostic tools for the early detection of WNV infection.

WNV is an enveloped, single-stranded positive-sense RNA virus of the genus Flavivirus (family Flaviviridae), with an ~11 kb genome lacking a 3′ poly (A) tail and encoding three structural (capsid, envelope, and premembrane) and seven non-structural (NS1, NS2A, NS2B, NS3, NS4A, NS4B, and NS5) proteins that collectively mediate viral entry, assembly, replication, and antagonism of host innate immunity [14,15,16,17,18,19]. Taxonomically, WNV has been divided into nine lineages [20], but most human outbreaks of WNND are caused by lineages 1 and 2. Lineage 1 exists worldwide and comprises clade 1a, isolated from Europe, Africa, and the Americas, and clade 1b (Kunjin virus), largely restricted to Oceania and only rarely associated with neurological disease [21,22,23]. Lineage 2, originally reported in Africa and Europe, has been considered less pathogenic than lineage 1, yet has nonetheless been responsible for severe neuroinvasive infections in birds, horses, and humans in South Africa and Europe [20,24,25].

For WNV diagnosis, the nucleic acid amplification tests (NAATs), such as quantitative reverse-transcription polymerase chain reaction (qRT-PCR) or RT-PCR, are mainly used to detect extracted viral RNA from blood or cerebrospinal fluid (CSF) [26]. However, these traditional methods often face limitations in terms of time, sensitivity, specificity, risk of contamination, high cost, required professional labor, and the need for sophisticated laboratory infrastructure. Owing to these challenges, the application of the conventional methods in low- and middle-income countries (LMIC) is difficult for detecting WNV within the early stages. Therefore, there is a need for WNV diagnostic platforms that retain high analytical performance while being compatible with simple, instrument-light workflows suitable for point-of-care testing.

Among recent developments in diagnostic technologies, the DNA endonuclease-targeted CRISPR trans reporter (DETECTR), a novel diagnostic tool that uses isothermal amplification with clustered regularly interspaced short palindromic repeats (CRISPR) and the CRISPR-associated (Cas) system, is of great interest [27]. Nucleic acids are amplified via recombinase polymerase amplification (RPA), one of the isothermal amplification techniques, as an alternative to PCR [28]. CRISPR-Cas is a member of the adaptive immune systems of bacteria and archaea; in particular, Cas12a from Lachnospiraceae bacterium (LbaCas12a) plays a role as a trans-cleavage activity when it recognizes target DNA with a T-nucleotide-rich protospacer-adjacent motif (PAM) sequence complementary to guide RNA (gRNA) [29]. Following these procedures, detection is finally performed through both fluorescence assay and lateral flow assay (LFA) (Figure S1). Since DETECTR has been introduced, diverse viruses, including severe acute respiratory syndrome coronavirus 2 (SARS-CoV-2), human papilloma viruses (HPVs), influenza A and B viruses (IAVs and IBVs), severe fever with thrombocytopenia syndrome virus (SFTSV), and Japanese encephalitis virus (JEV), have been successfully detected [27,30,31,32,33,34]. Despite these successful applications, a DETECTR-based molecular diagnostic assay targeting West Nile virus (WNV) has not yet been reported. Hence, 2-Step DETECTR, a rapid, sensitive, and field-applicable diagnostic method for detecting WNV by grafting DETECTR with a fluorescence assay or LFA, was developed in this study. Based on this conventional 2-Step format, we next designed a 1-Step WNV DETECTR that combines amplification and Cas12a-mediated detection in a single tube, referred to in previous reports as one-tube DETECTR, to simplify the workflow for potential point-of-care use. However, in practical point-of-care settings, performing both reactions within the same compartment may still impose limitations, including tube handling and an increased risk of contamination. To address these limitations, we therefore proposed a novel 1-Step with Filter WNV DETECTR that maintains a one-tube assay but physically separates the RT-RPA mixture from the LbaCas12a–gRNA–reporter solution using a filter insert, with the specific objective of reducing contamination risk and improving user-friendliness for field applications. In this study, these three DETECTR formats were comparatively evaluated in terms of analytical sensitivity, assay time, and operational simplicity to clarify the additional value of the 1-Step with Filter format for WNV point-of-care diagnosis.

## 2. Materials and Methods

### 2.1. Cells and Viruses

To produce lentivirus pseudotyped with WNV1a capsid gene (nucleotide 156 to 256) (GenBank accession number: KY703854) or WNV2 non-coding sequence between membrane and envelope genes (nucleotide 1462 to 1636) (GenBank accession number: AY532665.1) [35], the sequences were synthesized (Macrogen, Seoul, Republic of Korea) and amplified via PCR. Target gene amplification was performed with the following primers: forward (5′-GGGGACAAGTTTGTACAAAAAAGCAGGCTGGAGAGCCGGGCTGTCAATATGC-3′) and reverse (5′-GGGGACCACTTTGTACAAGAAAGCTGGGTAACTGCTCCTACGCTGGCGAT-3′) for WNV1a; forward (5′-GGGGACAAGTTTGTACAAAAAAGCAGGCTGGGGTGCGAAGTCCTTCCTGGTTC-3′) and reverse (5′-GGGGACCACTTTGTACAAGAAAGCTGGGTACAACGCACCTTCCTGCGACC-3′) for WNV2. Subsequently, PCR amplicons were respectively transmitted to pLenti CMV Puro DEST via BP and LR Gateway reactions (Invitrogen, Waltham, MA, USA) of GATEWAY cloning to generate pLenti CMV WNV1a gene vector and pLenti CMV WNV2 gene vector. The pLenti CMV Puro DEST (W118-1) construct was a gift from Eric Campeau and Paul Kaufman (Addgene #17452; http://n2t.net/addgene:17452 (accessed on 4 December 2025); RRID: Addgene_17452) [36].

For the production of lentivirus pseudotyped with the vectors, HEK293T cells were co-transfected with pMD2.G, pMDLG/pRRE, and pRSV-Rev, as well as either pLenti CMV WNV1a gene vector or pLenti CMV WNV2 gene vector using Omicsfect according to the manufacturer’s instructions (Omics Biotechnology, Seoul, Republic of Korea). pMD2.G, pMDLg/pRRE, and pRSV-Rev were gifts from Didier Trono (pMD2.5 Addgene plasmid #12259; http://n2t.net/addgene:12259 (accessed on 4 December 2025); RRID: Addgene_12259, pMDLg/pRRE Addgene plasmid #12251; http://n2t.net/addgene:12251 (accessed on 4 December 2025); RRID:Addgene_12251, pRSV-Rev Addgene plasmid # 12253; http://n2t.net/addgene:12253 (accessed on 4 December 2025); RRID:Addgene_12253) [37]. The culture medium was filtered through a 0.45 μm filter (Sartorius, Göttingen, Germany) to harvest the virus at 48 h post-transfection. While conducting transduction to perform titration, polybrene was added to the inoculum at a final concentration of 10 μg/mL. The lentiviral titer was estimated according to the Limiting Dilution–Colony Counting procedure at MD Anderson Cancer Center (https://www.mdanderson.org/).

Propagation and titration of the Influenza A virus (IAV) (A/Puerto Rico/8/1934), influenza B virus (IBV) (B/Brisbane/60/2008), and Japanese encephalitis virus genotype Ⅰ (JEV GⅠ) (NCCP43133) were conducted as described previously [31,38]. For the propagation and titration of the Seoul virus 80-39 (SEOV 80-39) (KBPV-VR-1009, Republic of Korea Bank for Pathogenic Viruses, Seoul, Republic of Korea), Dengue virus serotype 2 (DENV-2) (NCCP43255, National Culture Collection for Pathogens, Cheongju, Republic of Korea), and Zika virus (ZIKV) (NCCP43280, National Culture Collection for Pathogens, Cheongju, Republic of Korea), Vero E6 cells (Vero C1008) (Republic of Korean Collection for Type Cultures, Daejeon, Republic of Korea) were employed. The cells were cultured in Dulbecco’s modified Eagle’s medium (DMEM) (Hyclone, Logan, UT, USA) with 10% fetal bovine serum (FBS) (Capricorn, Ebsdorfergrund, Germany) and 1% penicillin and streptomycin (P/S) (Genomicbase, Seoul, Republic of Korea). The viruses were propagated in Vero E6 cells at passage 20, with DMEM containing 10% FBS and 1% P/S. Titration of the viruses was measured in 6-well culture plates. All experiments involving viruses were conducted under biosafety level 2 (BSL-2) conditions.

### 2.2. WNV RNA Synthesis

WNV RNA synthesized by in vitro transcription (IVT) was used to determine the sensitivity and specificity of the WNV DETECTR system. Both the WNV1a capsid gene (nucleotide 156 to 256) (GenBank accession number: KY703854) and the WNV2 non-coding sequence between the membrane and envelope genes (nucleotide 1462 to 1636) (GenBank accession number: AY532665.1) [35] were synthesized (Macrogen, Seoul, Republic of Korea) and amplified by PCR using Phusion^®^ High-Fidelity PCR Master Mix with HF Buffer (New England Biolabs, Ipswich, MA, USA) with specific primers containing a T7 promoter for each lineage (Table S1). The PCR amplicons were utilized in IVT reaction using the mMESSAGE mMACHINE T7 Transcription kit (Invitrogen, Waltham, MA, USA) to synthesize WNV RNAs. Purification of the synthesized RNAs was carried out using GeneJET RNA Cleanup and Concentration Micro Kit (Thermo Scientific™, Waltham, MA, USA). All of the copy numbers in this study were calculated by NEBioCalculator (version 1.17.4; https://nebiocalculator.neb.com (accessed on 4 December 2025)) based on the length and mass of RNA.

### 2.3. Viral RNA Extraction

Viral RNAs for WPV1a (lentivirus pseudotyped with WNV1a capsid gene), WPV2 (lentivirus pseudotyped with WNV2 non-coding sequence between membrane and envelope genes), CPV (lentivirus pseudotyped without any WNV genes), SEOV 80-39, IAV, IBV, JEV, DENV-2, and ZIKV were extracted to determine the specificity of WNV DETECTR using Punch-it™ NA-Sample Kit (Nanohelix, Daejeon, Republic of Korea) according to the manufacturer’s instructions. Moreover, to compare the lysis efficiency, viral RNAs of both WPV1a and WPV2 were extracted using Heating Unextracted Diagnostic Samples to Obliterate Nucleases (HUDSON) and AccuPrep^®^ Viral RNA Extraction Kit (Bioneer, Daejeon, Republic of Korea), according to the previous study and manufacturer’s instructions, respectively [39].

### 2.4. Designing RPA Primers and gRNAs for DETECTR

RPA primers and gRNAs for DECTECTR were designed based on the NCBI database. Nucleotide sequences of WNV isolate ArD76986/1990/SN (GenBank accession number: KY703854) and WNV strain B956 (GenBank accession number: AY532665.1) were employed to design the primers and gRNAs for WNV1a and WNV2, respectively.

**Figure 1:**
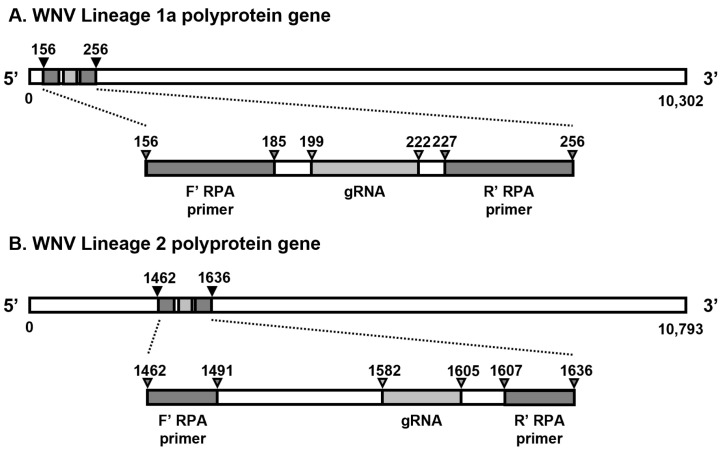
Diagrams of RPA primers and gRNAs for WNV DETECTR. (**A**) WNV lineage 1a; (**B**) WNV lineage 2.

Designing RPA primers was referred to the manufacturer’s manual of TwistAmp^®^ Basic Kit (TwistDx, Cambridge, UK) (Table 1, Figures S2A,B and S3). Lineage-specific RPA primer sets were used for viral RNA amplification via RT-RPA. For WNV1a, the forward primer sequence aligned with nucleotides 156 to 185, while the reverse primer sequence corresponded to nucleotides 227 to 256. For WNV2, the forward primer sequence aligned with nucleotides 1462 to 1491, while the reverse primer sequence corresponded to nucleotides 1607 to 1636 (Figure 1). The reverse complementary orientation was applied to all the reverse primers. The RPA primers were aligned with WNV lineages (Figures S2A,B and S3).

Designing gRNAs with the PAM sequence (TTTV, V is A, G, or C) to detect WNV lineage 1a and 2, respectively, was conducted based on the NCBI database (Table 2, Figures S2C,D and S4). WNV1a gRNA was targeted to nucleotides 199 to 222, and WNV2 gRNA was targeted to nucleotides 1582 to 1605 (Bioneer, Daejeon, Republic of Korea) (Figure 1). The reverse complementary orientation was applied to WNV1a gRNA. The gRNAs were aligned with WNV lineages (Figure S2C,D and S4).

### 2.5. Two-Step DETECTR

**Figure 2:**
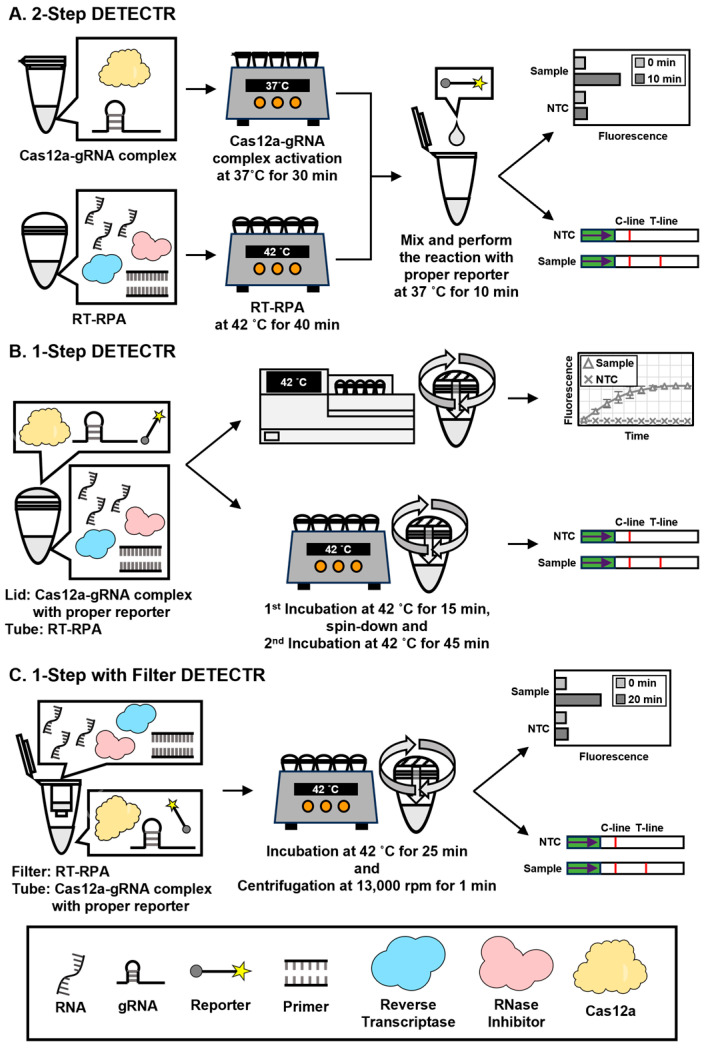
Schematic diagrams of 2-Step, 1-Step, and 1-Step with Filter DETECTR workflows. (**A**) 2-Step DETECTR; (**B**) 1-Step DETECTR; (**C**) 1-Step with Filter DETECTR.

The 2-Step DETECTR is a fundamental method that involves two distinct steps: amplification and activation. Each step is independently performed in separate tubes at different times and temperatures (Figure 2A).

To amplify viral RNA, RT-RPA was performed with the TwistAmp^®^ Basic Kit. The RT-RPA reaction mixture consisted of 29.5 μL of rehydration buffer, 2.4 μL of 10 μM forward and reverse primers, 1 μL of TOPscript™ Reverse Transcriptase (Enzynomics, Daejeon, Republic of Korea), 1 μL of RNase inhibitor (Enzynomics, Daejeon, Republic of Korea), and 2.5 μL of 280 mM Magnesium Acetate (MgOAc) following the manufacturer’s instructions. Samples and nuclease-free water were added to the reaction mixture up to a final volume of 50 μL. In this study, 10 μL of the sample was added to yield 1 × 10^3^ to 1 × 10^2^ RNA copies, and the final RT-RPA reaction mixture was incubated at 42 °C for 40 min.

The LbaCas12a trans-cleavage assay was implemented as described previously [30,31,34]. To activate the LbaCas12a-gRNA complex, EnGen^®^ Lba Cas12a (Cpf1, LbaCas12a) (New England Biolabs, Ipswich, MA, USA) and gRNA were added to 1x NEB 2.1 buffer to final concentrations of 50 nM and 62.5 nM, respectively, and then the reaction mixture was incubated at 37 °C for 30 min.

To measure fluorescence saturation, 80 μL of 1x NEB 2.1 buffer, 18 μL of LbaCas12a-gRNA complex, 2 μL of 10 μM FQ-labeled reporter (/56-FAM/TTATT/3IABkFQ/; Integrated DNA Technologies, Coralville, IA, USA), and 2 μL of RT-RPA products were added directly to each well of 96-well microplates. Fluorescence results were obtained at 0, 10, and 20 min using a GloMax^®^ Discover Microplate Reader (Promega Corporation, Madison, WI, USA) (λex, 485 nm; λem, 535 nm). In the case of results observed at 10 and 20 min, the 96-well microplates were incubated at 37 °C prior to processing the fluorescence assay.

For LFA, 22 μL of 1x NEB 2.1 buffer, 36 μL of LbaCas12a-gRNA complex, 2 μL of 10 μM lateral flow cleavage reporter (/56-FAM/TTATT/3Bio/; Integrated DNA Technologies), and 20 μL of RT-RPA products were mixed in a 1.5 mL tube and incubated at 37 °C for 10 min. After that, Milenia HybriDetect 1 lateral flow strips (Milenia, Giesesen, Germany) were placed in the incubated sample tubes following the manufacturer’s instructions, and the results were evaluated after 2 min.

The LoD in both fluorescence assay and LFA was defined as the lowest RNA copy number at which the mean ΔF (F_10min_ − F_0min_; F, Fluorescence saturation value) could be distinguished from that of the NTC (two-sample *t*-test, *p* < 0.05).

### 2.6. 1-Step DETECTR

The 1-Step DETECTR is a simplified method that integrates amplification and activation steps in the same tube with the same time and temperature conditions. The 1-Step DETECTR process was designed as described previously [31,40,41]. The method was composed of two separate components in a tube. The RT-RPA reaction mixture was located in the tube to amplify viral RNA, and simultaneously, the LbaCas12a-gRNA complex and reporter were placed in the lid to activate the detection system (Figure 2B).

For amplification, the RT-RPA reaction mixture containing 29.5 μL of rehydration buffer, 2.4 μL of 10 μM forward and reverse primers, 1 μL of TOPscript™ Reverse Transcriptase, and 1 μL of RNase inhibitor was divided into two tubes. Samples and nuclease-free water were added to the reaction mixture up to a volume of 27.25 μL. In this study, 5 μL of the sample was added to yield 1 × 10^4^ to 1 × 10^1^ RNA copies. RPA powder resuspended in 13 μL of nuclease-free water was separated into 6.5 μL aliquots. Subsequently, the aliquots were added to each tube together with 1.25 μL of 280 mM MgOAc.

For the activation of the LbaCas12a-gRNA complex to use in the fluorescence assay, 2.5 μL of 10x NEB 2.1 buffer, with 1 μL of 1 μM LbaCas12a, 0.5 μL of 1 μM gRNA, and 1 μL of 10 μM FQ-labeled reporter was placed in the lid of the same tubes. Meanwhile, 29 μL of 10x NEB 2.1 buffer with 8 μL of 1 μM LbaCas12a, 1 μL of 1 μM gRNA, and 1 μL of 10 μM lateral flow cleavage reporter was placed in the lid of the same tubes for activation to use in LFA. The tubes were closed carefully to ensure no droplets of the mixture.

To measure fluorescence saturation, an Axxin T16-ISO instrument (Axxin, Fairfield, Australia) was employed. Tubes were incubated at 42 °C for 15 min, briefly spun down to transfer the mixture from the lid to the tubes, then further incubated at 42 °C for 45 min. Fluorescence results were obtained in real time every 5 min during the 2nd incubation.

For LFA, tubes were incubated at 42 °C for 15 min, briefly spun down to mix the mixture from the lid with the tubes, then further incubated at 42 °C for 45 min. After that, Milenia HybriDetect 1 lateral flow strips were placed in the incubated sample tubes following the manufacturer’s instructions, and the results were evaluated after 2 min. The LoD was defined in the same way as described in the Two-Step DETECTR method (Section 2.5).

### 2.7. 1-Step with Filter DETECTR

The 1-Step with Filter DETECTR is similar to the 1-Step DETECTR, but improves on its limitations. The method was composed of two components separated by using a 0.22 μm filter (SPL, Pocheon, Republic of Korea). The RT-RPA reaction mixture was located in the filter to amplify viral RNA, and simultaneously, the LbaCas12a-gRNA complex and reporter were placed in the tube to activate the detection system (Figure 2C).

For amplification, the RT-RPA reaction mixture consisted of 29.5 μL of rehydration buffer, 2.4 μL of 10 μM forward and reverse primers, 1 μL of TOPscript™ Reverse Transcriptase, 1 μL of RNase inhibitor, and 2.5 μL of 280 mM MgOAc following the manufacturer’s instructions. Samples and nuclease-free water were added to the reaction mixture up to a final volume of 50 μL. In this study, 10 μL of the sample was added to yield 1 × 10^3^ to 1 × 10^2^ RNA copies. The amplification reaction mixture was transferred to the filter after combining the filters with the tubes.

For activation, the LbaCas12a and gRNA were added to 1x NEB 2.1 buffer to yield final concentrations of 50 nM and 62.5 nM, respectively. Furthermore, 1 μL of 10 μM FQ-labeled reporter or lateral flow cleavage reporter was mixed for the following detection step. Finally, 60 μL of the reaction mixture was placed into the tubes prior to combining the filter.

For the fluorescence assay, the tubes were incubated at 42 °C for 25 min and then centrifuged to transfer the RT-RPA reaction mixture from the filter to the tube at 13,000 rpm for 1 min. The final mixture was transferred to each well of 96-well microplates, and fluorescence saturation was measured at 0 and 20 min using a GloMax^®^ Discover Microplate Reader. In the case of the results at 20 min, the 96-well microplates were incubated at 42 °C prior to processing the fluorescence assay.

To evaluate the LFA result, tubes were incubated at 42 °C for 25 min, centrifuged to transfer the RT-RPA reaction mixture from the filter to the tube at 13,000 rpm for 1 min, and then further incubated at 42 °C for 20 min. After that, Milenia HybriDetect 1 lateral flow strips were placed in the incubated sample tubes following the manufacturer’s instructions, and the results were evaluated after 2 min. The LoD was defined in the same way as described in the Two-Step DETECTR method (Section 2.5).

### 2.8. Gel Electrophoresis

To evaluate the efficiency of lysis methods, gel electrophoresis was carried out to quantitatively analyze the outcome of the RT-RPA product amplified with extracted viral RNA using each lysis method. The RT-RPA amplicons were mixed with 6x DNA loading buffer to yield a final concentration of 1x, and the mixture was loaded in a 0.5x Tris-boric acid-EDTA (TBE) buffer 2.5% agarose gel stained with 0.5 μg/mL EtBr. The agarose gel was exposed to UV to evaluate the size of the RT-RPA amplicon after running for 80 min at 130 V.

## 3. Results

### 3.1. Establishment of WNV DETECTR

To establish a CRISPR-Cas12a-based diagnostic assay for WNV, we first identified lineage-specific target regions within the WNV genome and designed DETECTR components accordingly. Reverse transcription–recombinase polymerase amplification (RT-RPA) primers were selected to amplify the capsid gene of WNV lineage 1a and the non-coding region between the membrane and envelope genes of lineage 2 (Figure 1, Table 1). Sequence alignment of the available WNV isolates showed that these regions are highly conserved among lineage 1a and 2 strains that are major causes of neurological disease, with only a few strains (KC601756 and GQ379161 in WNV1a, and GQ903680 in WNV2) harboring mismatches within the primer and gRNA binding sites (Figures S3 and S4) [42,43,44]. Based on these conserved regions, we generated lineage-specific RT-RPA primers and Cas12a guide RNAs (gRNAs), thereby establishing the basic WNV DETECTR architecture. Using this configuration, RT-RPA products were subsequently subjected to trans-cleavage by an activated LbaCas12a–gRNA complex in the presence of reporter oligonucleotides, enabling downstream signal readout. The ssDNA-fluorophore (FAM) quencher (FQ)-labeled reporter was employed for fluorescence assay; meanwhile, the ssDNA-FAM-Biotin (FB) reporter was used for LFA (Figure 2A). Building on the conventional 2-Step WNV DETECTR, which requires separate amplification and detection steps, we then developed simplified assay formats. First, we combined the amplification and detection reactions into a single tube to generate a 1-Step WNV DETECTR format, in which the RT-RPA mixture and the LbaCas12a–gRNA complex were spatially separated within the same reaction tube (Figure 2B). Finally, to further reduce handling steps and mitigate the risk of contamination, we established a 1-Step with Filter WNV DETECTR system that allows for RT-RPA and Cas12a activation to proceed within a single tube while physically separating the amplification mixture from the LbaCas12a–gRNA–reporter solution using a filter insert (Figure 2C). These stepwise developments provided three distinct WNV DETECTR formats that were subsequently evaluated for analytical sensitivity and assay performance in the following sections.

### 3.2. Determination of Limit of Detection (LoD) of 2-Step WNV DETECTR

**Figure 3:**
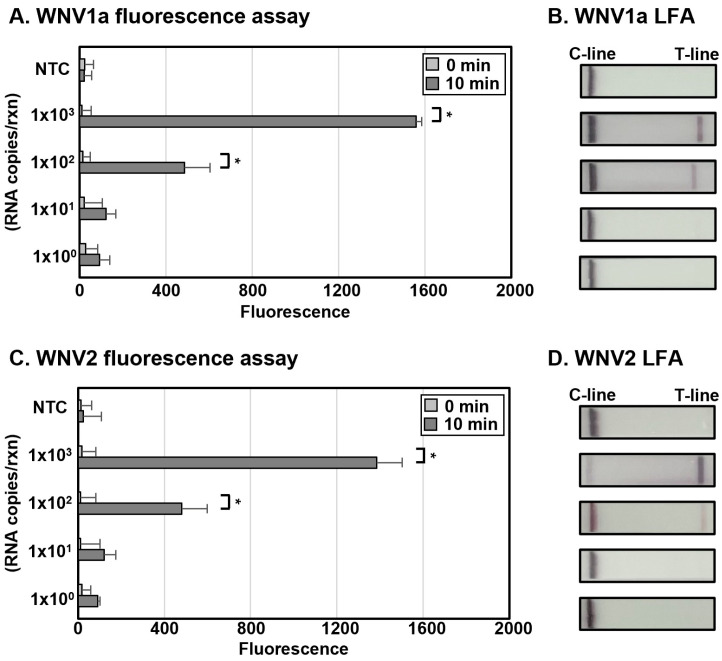
Sensitivity of 2-Step WNV DETECTR. Different concentrations of RNA (1.0 × 10^3^ to 1.0 × 10^0^ RNA copies per reaction) were used to amplify viral nucleic acids via RT-RPA with primer sets specific for each lineage. Fluorescence saturation was measured within 10 min after reaction of the LbaCas12a-gRNA complex with the RT-RPA products at 37 °C (**A**,**C**). LFA results were evaluated at 2 min after the strip was activated with the sample (**B**,**D**). Error bars represent mean ± s.e. (*n* = 3 replicates; * *p* < 0.05 between 0 min and 10 min samples, two-sample *t*-test). NTC, non-template control; C-line, control line; T-line, test line.

To establish the sensitivity of 2-Step WNV DETECTR, IVT was employed to synthesize RNAs of the two WNV lineages, which were used for RT-RPA. The synthesized RNAs were treated with an RT-RPA reaction mixture by 10-fold serial dilution using RNase-free distilled water (1.0 × 10^3^ to 1.0 × 10^2^ RNA copies per reaction). In both fluorescence assay and LFA, the 2-Step WNV DETECTR was effectively available to detect the synthesized WNV RNAs by IVT up to 1.0 × 10^2^ RNA copies per reaction (Figure 3). The LoD of the 2-Step WNV DETECTR using WNV1a and WNV2 in vitro-transcribed RNA fragments was 1.0 × 10^2^ RNA copies per reaction. Compared to the conventional RT-PCR method, this technique is approximately 50 times less sensitive (2 RNA genome copies per reaction by qRT-PCR), though it is a sufficiently sensitive approach without cross-reactivity, with a short amplification process that is 68 min shorter than RT-PCR, and without the need for expensive equipment, like a thermocycler [26].

### 3.3. Determination of LoD of 1-Step WNV DETECTR

**Figure 4:**
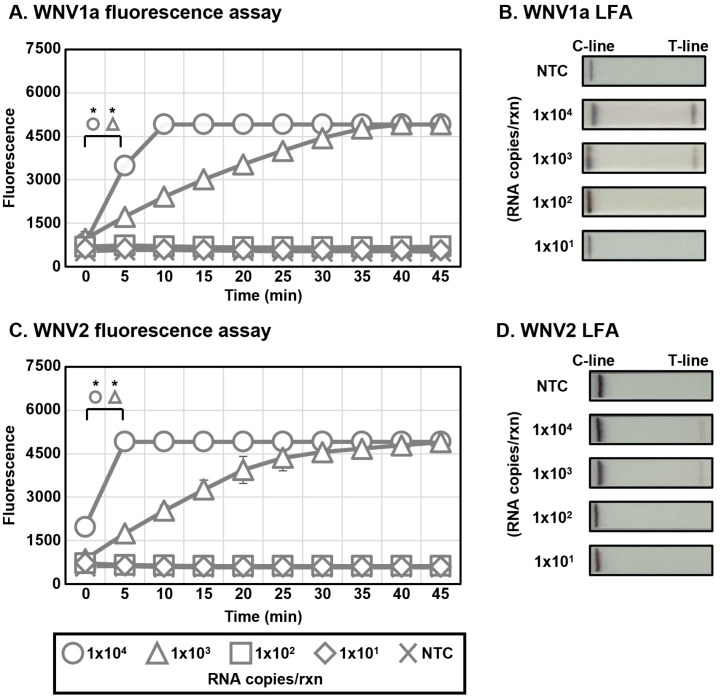
Sensitivity of 1-Step WNV DETECTR. Different concentrations of RNA (1.0 × 10^4^ to 1.0 × 10^1^ RNA copies per reaction) were used to amplify viral nucleic acids via RT-RPA with primer sets specific for each lineage. Fluorescence saturation was measured in real time by Axxin T16-ISO (**A**,**C**), and LFA results were evaluated at 2 min after the strip was activated with the sample (**B**,**D**). Error bars represent mean ± s.e. (*n* = 3 replicates; * *p* < 0.05 between 0 min and 5 min samples, two-sample *t*-test). NTC, non-template control; C-line, control line; T-line, test line.

For evaluating the sensitivity of 1-Step WNV DETECTR, RNAs of the two WNV lineages for RT-RPA were synthesized via IVT. Synthesized RNAs were added to an RT-RPA reaction mixture by 10-fold serial dilution using RNase-free distilled water (1.0 × 10^4^ to 1.0 × 10^1^ RNA copies per reaction). The method detected synthesized WNV RNAs by IVT up to 1.0 × 10^3^ RNA copies per reaction in both the fluorescence assay and LFA (Figure 4). Therefore, the LoD of the 1-Step WNV DETECTR using WNV1a and WNV2 in vitro-transcribed RNA fragments was 1.0 × 10^3^ RNA copies per reaction. Consequently, the 1-Step WNV DETECTR was less sensitive than the 2-Step technique by about 10-fold. Even though the total processing time increased by 10 min compared to the 2-Step method, the workflow was simplified by integrating amplification and activation processes into a single tube.

### 3.4. Determination of LoD of 1-Step with Filter WNV DETECTR

**Figure 5:**
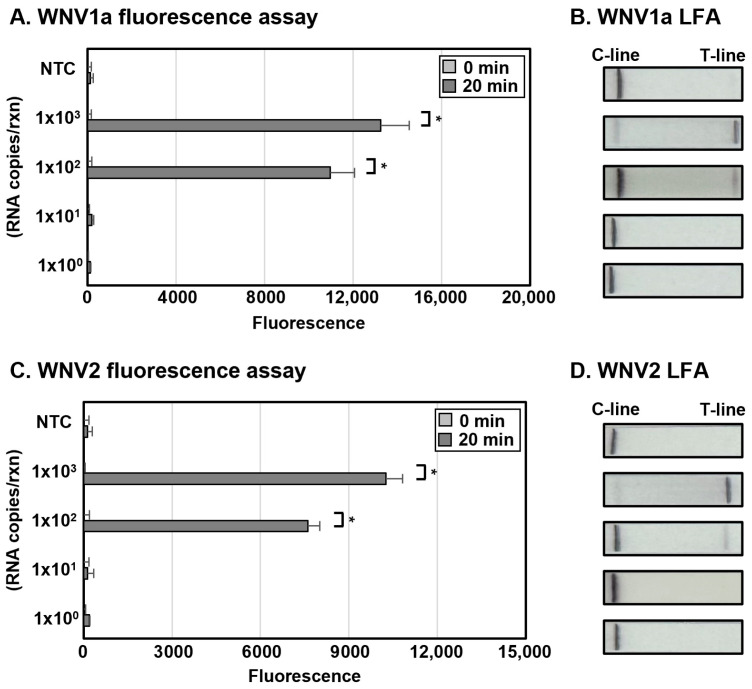
Sensitivity of 1-Step with Filter WNV DETECTR. Different concentrations of RNA (1.0 × 10^3^ to 1.0 × 10^0^ RNA copies per reaction) were used to amplify viral nucleic acids via RT-RPA with primer sets specific for each lineage. Fluorescence saturation was measured within 20 min after reaction of the LbaCas12a-gRNA complex with the RT-RPA products at 42 °C (**A**,**C**). LFA results were evaluated at 2 min after the strip was activated with the sample (**B**,**D**). Error bars represent mean ± s.e. (*n* = 3 replicates; * *p* < 0.05 between 0 min and 20 min samples, two-sample *t*-test). NTC, non-template control; C-line, control line; T-line, test line.

To determine the sensitivity of the 1-Step with Filter WNV DETECTR, WNV RNAs of each lineage for RT-RPA were synthesized by IVT, and the products were added with 10-fold serial dilution using RNase-free distilled water to an RT-RPA reaction mixture (1.0 × 10^3^ to 1.0 × 10^2^ RNA copies per reaction). The synthesized WNV RNAs by IVT were competently detected up to 1.0 × 10^2^ RNA copies per reaction in both the fluorescence assay and LFA (Figure 5). Therefore, the LoD of the 1-Step with Filter WNV DETECTR using WNV1a and WNV2 in vitro-transcribed RNA fragments was 1.0 × 10^2^ RNA copies per reaction. Overall, the 1-Step with Filter WNV DETECTR had a similar sensitivity to the 2-Step WNV DETECTR, and more than the 1-Step WNV DETECTR by about 10-fold in less time. The total processing time was reduced by 5 min compared to the 2-Step method and by 15 min compared to the 1-Step method. Additionally, like the 1-Step method, it minimized the potential for contamination as well as simplified the workflow. The characteristics of the WNV DETECTR assays developed in this study and the reference qRT-PCR are summarized and compared in Table 3.

### 3.5. Determination of Efficiency of Lysis Methods

**Figure 6:**
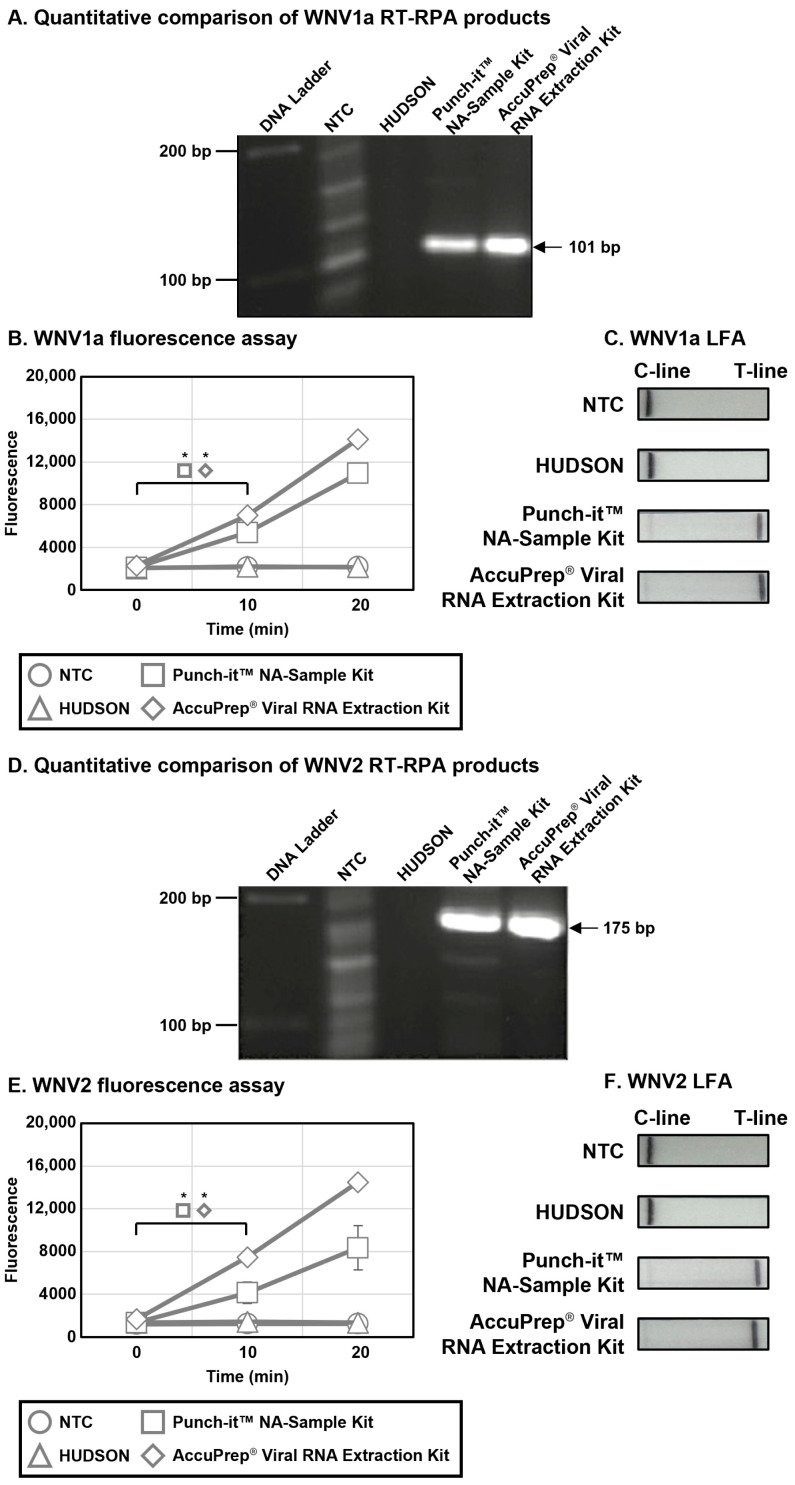
Efficiency comparison of lysis methods. Viral RNAs for WPV1a and WPV2 (1.0 × 10^4^ CFU per mL) were extracted using HUDSON, Punch-it™ NA-Sample Kit (Nanohelix, Daejeon, Republic of Korea) and AccuPrep^®^ Viral RNA Extraction Kit (Bioneer, Daejeon, Republic of Korea), and amplified using RT-RPA. RT-RPA amplicons were analyzed by agarose gel electrophoresis (**A**,**D**) and 2-Step WNV DETECTR with fluorescence readout (**B**,**E**) or LFA (**C**,**F**). Error bars represent mean ± s.e. (*n* = 3 replicates; * *p* < 0.05 between 0 min and 10 min samples, two-sample *t*-test). CFU, colony forming unit; PFU, plaque forming unit; DNA Ladder, molecular weight marker of 100 bp DNA Ladder (Solis Biodyne); NTC, non-template control; C-line, control line; T-line, test line.

To compare the efficiency of lysis methods, viral RNAs for WPV1a and WPV2 (1.0 × 10^4^ CFU per mL) were extracted using HUDSON, a Punch-it™ NA-Sample Kit, and an AccuPrep^®^ Viral RNA Extraction Kit and amplified using RT-RPA with primer sets specific to each lineage. At first, the synthesized products were quantitatively evaluated via agarose gel electrophoresis using EtBr. The NTC lane, which contained only distilled water in the RT-RPA reaction, showed bands of various sizes, whereas the lane with RNA extracted using the AccuPrep^®^ Viral RNA Extraction Kit, a gold standard for viral nucleic acid extraction, showed a single strong band at the expected product size. Concerning the lane of the Punch-it™ NA-Sample Kit, the same results as the lane of the AccuPrep^®^ Viral RNA Extraction Kit were obtained; however, no bands were observed in the lane of the HUDSON (Figure 6A,D). Next, the RT-RPA amplicons of each lysis method were detected by 2-Step WNV DETECTR combined with either fluorescence assay or LFA, utilizing gRNAs specific for each lineage, to confirm practical efficiency in DETECTR. In both assays, samples processed with the Punch-it™ NA-Sample Kit and the AccuPrep^®^ Viral RNA Extraction Kit showed a significant difference within 10 min, while the NTC and HUDSON samples did not show such a difference (Figure 6B,C,E,F). Although the fluorescence saturation of RT-RPA products obtained using the Punch-it™ NA-Sample Kit in both assays was slightly lower than that obtained using the AccuPrep^®^ Viral RNA Extraction Kit, it was sufficient to use for viral RNA extraction. In conclusion, the efficiency of Punch-it™ NA-Sample Kit as a lysis method was similar to AccuPrep^®^ Viral RNA Extraction Kit.

### 3.6. Specificity of WNV DETECTR

**Figure 7:**
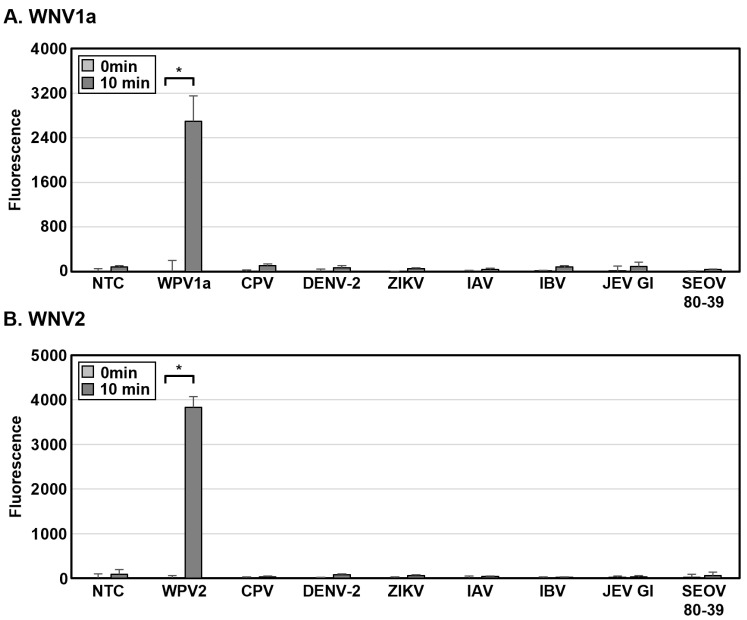
Specificity of WNV DETECTR. (**A**,**B**) Viral RNAs from WPV1a (lentivirus pseudotyped with WNV1a capsid gene), WPV2 (lentivirus pseudotyped with WNV2 non-coding sequence between membrane and envelope genes), CPV (lentivirus pseudotyped without any WNV genes) (1.0 × 10^4^ CFU), and from SEOV 80-39, IAV, IBV, JEV, DENV-2, and ZIKV (1.0 × 10^4^ PFU) were extracted using Punch-it™ NA-Sample Kit (Nanohelix, Daejeon, Republic of Korea) and amplified by RT-RPA. RT-RPA products were analyzed by 2-Step WNV DETECTR with fluorescence readout. Fluorescence saturation was measured within 10 min after incubation of the LbaCas12a-gRNA complex with the RT-RPA products at 37 °C. Error bars represent mean ± s.e. (*n* = 3 replicates; * *p* < 0.05 between 0 min and 10 min samples, two-sample *t*-test). CFU, colony-forming unit; PFU, plaque-forming unit; WPV1a, WNV1a pseudovirus; WPV2, WNV2 pseudovirus; CPV, control pseudovirus; NTC, non-template control.

To evaluate the WNV DETECTR, lentiviruses pseudotyped with WNV1a capsid gene or WNV2 non-coding sequence between membrane and envelope genes were generated. Viral RNAs for WPV1a (lentivirus pseudotyped with WNV1a capsid gene) and WPV2 (lentivirus pseudotyped with WNV2 non-coding sequence between membrane and envelope genes) were extracted using Punch-it™ NA-Sample Kit according to the manufacturer’s instructions. Extracted viral nucleic acids were detected following 2-Step WNV DETECTR combined with a fluorescence assay utilizing gRNAs specific for each lineage. CPV (lentivirus pseudotyped without any WNV genes), SEOV 80-39, IAV, IBV, JEV, DENV-2, and ZIKV were used as controls to determine the specificity of WNV DETECTR. Their viral RNAs were also extracted using a Punch-it™ NA-Sample Kit and applied to the same detection method. The titers of WPV1a, WPV2, and CPV were 1.0 × 10^4^ colony-forming units (CFU) per mL, and SEOV 80-39, IAV, IBV, JEV, DENV-2, and ZIKV were 1.0 × 10^4^ plaque-forming units (PFU) per mL. In the fluorescence assay for both lineages of WNV, WNV DETECTR successfully detected WPV1a and WPV2 without cross-reactivity with CPV, SEOV 80-39, IAV, IBV, JEV, DENV-2, and ZIKV (Figure 7).

## 4. Discussion

In this study, we developed a rapid and sensitive CRISPR-Cas12a-based diagnostic method for WNV lineage 1a and 2 without cross-activity. Moreover, we steadily improved the de novo WNV diagnostic format, focusing on rapidness, sensitivity, simplicity, low risk of contamination, and field availability, from the 2-Step to the 1-Step, and ultimately, the 1-Step with Filter system. All these techniques successfully detected in vitro-transcribed WNV lineage 1a and 2 viral RNA (Figure 3, Figure 4 and Figure 5), and the high specificity of the newly developed WNV diagnostic method was proved by analysis with viral RNA extracted from a lenti-pseudovirus possessing a genome identical to that of WNV as a positive-sense RNA virus (Figure 6 and Figure 7). For evaluating the LoD of the methods using DETECTR, the measurement of fluorescence saturation was achieved by fluorescence assay. The LFA, which is an optimal detection method for the purpose of this study due to several advantages, such as low cost, simplification, rapidness, and portability, was employed as well [45]. In particular, LFA would be a competitive detection method in not only advanced countries, but also LMICs and environments with relatively insufficient medical infrastructure. The analytical and practical differences between each DETECTR method and qRT-PCR are organized and compared in Table 3. Although the LoD of WNV DETECTR in this study was higher than that of qRT-PCR (2 RNA genome copies per reaction) [26], it was sufficiently sensitive with a rapid amplification step that takes 61–63 min, less than RT-PCR (based on 1-Step with Filter WNV DETECTR), and simplifying the procedures by removing sophisticated experimental machines, such as a thermocycler, in the overall process (Figure 2). In particular, the 1-Step with Filter WNV DETECTR has not only the merits of all DETECTR formats, but also high potential when the method is commercialized. In previous techniques, the necessity of pushing down the solution using a special instrument existed, but the mixture in 1-Step with Filter DETECTR may be easily passed by portable piston pressure. As a result, our final goal of WNV DETECTR, the 1-Step with Filter WNV DETECTR, would be able to become a novel, effective, and appropriate field diagnosis tool.

For effective field diagnostics, rapid and simple nucleic acid extraction is essential. Current viral nucleic acid extraction methods include thermal and chemical treatments, as well as commercial membrane-based kits, which are considered the gold standard, using silica filters with chaotropic salts. To evaluate these methods, we compared the extraction efficiency of HUDSON and a viral RNA extraction kit. Moreover, we assessed the Punch-it™ NA-Sample Kit, which, unlike traditional commercial kits, extracts nucleic acids based on a paper chromatographic method, utilizing differences in solubility for the mobile phase (solvent) and affinity for the stationary phase (paper). Therefore, the WNV RNA was extracted using not HUDSON, which is known as an easy and simple method for viral nucleic acid extraction in a previous study, but a Punch-it™ NA-Sample Kit to determine the specificity of WNV DETECTR. HUDSON required to set an optimal temperature and concentration of reagents for each virus, and it was relatively difficult to apply in the field due to the necessity of a heat instrument. In the case of Punch-it™ NA-Sample Kit, it worked easily to isolate nucleic acids from various samples within 5 min without requiring any equipment, so it may offer positive effects, such as portability and accessibility, to DETECTR as diagnostic tools in the field when brought to commercialization. To estimate the efficiency of viral RNA extraction of the Punch-it™ NA-Sample Kit compared with the lysis methods, the RT-RPA amplicons using extracted viral RNAs by each lysis technique were detected by agarose gel electrophoresis and 2-Step WNV DETECTR combined with fluorescence assay or LFA (Figure 6). Given the results, Punch-it™ NA-Sample Kit was competitive compared with HUDSON and the viral RNA Extraction Kit in lysis. As a result, Punch-it™ NA-Sample Kit may be an efficient and useful technique combined with DETECTR as a novel improvement in a diagnostic tool in the future, if DETECTR were to become commercialized.

Overall, the WNV DETECTR is expected to be sufficiently sensitive to the actual detection of WNV, because it accurately detected both in vitro-transcribed synthetic RNA and viral RNA extracted from a lenti-pseudovirus mimicking the WNV genome, without any cross-reactivity. Nevertheless, it still had a few challenges and requires further investigation with clinical samples in the future. For instance, a few WNV strains showed mismatches with RPA primers/gRNAs. However, previous studies have reported that RPA is relatively tolerant to multiple mismatches within the primer- and template-binding regions. Especially, when these are not clustered at the 3′ termini, up to 5–9 mismatches can have little impact on assay performance [46]. In addition, several reports have indicated that Cas12a also displays a certain degree of mismatch tolerance between the crRNA spacer and the target sequence. Cas12a can retain robust cis-cleavage and trans-cleavage activity in the presence of single or even multiple mismatches, with the functional outcome depending on the position and number of mismatches [47]. Therefore, the alignment of currently available sequences, based on the known mismatch tolerance of RPA and CRISPR/Cas12a system-based assays, indicated that our RPA primers and Cas12a gRNAs are predicted to detect 94.4% of WNV lineage 1a and 97.0% of lineage 2 sequences. This indicates that the assay is expected to cover the vast majority of epidemiologically relevant WNV strains and provides a broad detection range, although a few numbers of highly divergent strains may not be efficiently detected and would require future primer/gRNA re-design if they emerge in the field. In terms of validation with clinical samples, there is a report noting that extracellular RNA can be employed as a positive control for RPA and DETECTR [32]; therefore, applying DETECTR on viruses extracted from patient CSF or blood is essential for determining the diagnostic efficiency of them, and establishing whether the viral RNAs are easily extracted using a Punch-it™ NA-Sample Kit on clinical samples. Lastly, the present WNV DETECTR assay achieves an LoD approximately 50 times higher than that of qRT-PCR, and therefore, continued efforts will be required to improve the sensitivity/LoD of the WNV DETECTR platform through the optimization of assay conditions.

In summary, we have accomplished that WNV DETECTR could be employed as a novel diagnostic tool that has a relatively short processing time, is more sensitive, and is lineage-specific. The sensitivity of the 2-Step WNV DETECTR was sufficiently high, saving processing time and cost relative to other conventional methods. Moreover, the 1-Step WNV DETECTR was designed. Even though it had lower sensitivity than the 2-Step WNV DETECTR, the technique could be applied to perform a quick test under single temperature conditions. To improve the method, the 1-Step with Filter WNV DETECTR was designed to minimize contamination and simplify the procedure with high sensitivity. Also, to achieve the same purpose, a Punch-it™ NA-Sample Kit, which has high optimization for a field type, was employed for viral RNA extraction. Hence, the 2-Step DETECTR, 1-Step DETECTR, and 1-Step with Filter DETECTR demonstrate significant advantages, including rapid and simple amplification and detection, coupled with high target susceptibility and sensitivity in various settings, such as areas with limited medical infrastructure or field conditions. Considering these positive aspects, these methods have high potential to emerge as novel and effective diagnostic tools, offering an alternative to conventional methods and overcoming financial and technical limitations.

## Figures and Tables

**Table 1:** RPA primers for WNV DETECTR.

Lineage	Primer	Sequence
WNV1a	1a_RT-RPA_F	GTTCTTCAGGTTCACAGCAATTGCTCCGAC
	1a_RT-RPA_R	TCTTAAAACTCAGAAGGTGTTTCATCGCTG
WNV2	2_RT-RPA_F	TACGTTATGTCAGTTGGTGCGAAGTCCTTC
	2_RT-RPA_R	CTTCCTGCGACCCTAGAGCCACAACAGATT

**Table 2:** gRNAs for WNV DETECTR.

Lineage	Primer	PAM	Sequence
WNV1a	1a_gRNA	TTTG	TTCACACCTCTCCATCGATC
WNV2	2_gRNA	TTTG	AAGAACCTCATGCCACCAAA

**Table 3:** WNV DETECTR performance comparison with qRT-PCR.

Method	Target Sequence	Time (Minutes)	Specificity (+/−) *	LoD (RNA Copies/Reaction)	Merit	Reference
qRT-PCR	5′-UTR ^1^ NS2A ^2^	108	−	2.0	High sensitivity	[26]
2-Step DETECTR	C gene ^3^ (lineage 1a) M-E NCR ^4^ (lineage 2)	50–52	−	1.0 × 10^2^	Rapid, Low cost, Instrument-light	In this study
1-Step DETECTR		60–62	−	1.0 × 10^3^	High simplicity	
1-Step with Filter DETECTR		45–47	−	1.0 × 10^2^	Low risk of contamination, Rapid, Sensitive, Field-Optimized	

* +: Cross-reactivity; −: No cross-reactivity. ^1^ 5′-UTR: 5′-untranslated region. ^2^ NS2A: Nonstructural protein 2A gene. ^3^ C gene: Capsid gene. ^4^ M-E NCR: Non-coding region between membrane and envelope genes.

## Data Availability

All data and materials supporting the conclusions are described and included in this manuscript.

## Supplementary Materials

The following supporting information can be downloaded at: https://www.mdpi.com/article/10.3390/bios15120807/s1, Table S1: Primers for in vitro transcription; Figure S1: Schematic diagrams of DETECTR and LFA; Figure S2: RPA Primer and gRNA alignments for WNV DETECTR; Figure S3: Sequence alignments of WNV based on RPA Primer; Figure S4: Sequence alignments of WNV based on gRNA.

## Abbreviations

The following abbreviations are used in this manuscript:

WNV	West Nile virus
WNF	West Nile fever
WNND	West Nile neuroinvasive disease
WNM	West Nile meningitis
WNE	West Nile encephalitis
WNP	West Nile poliomyelitis
NAATs	Nucleic acid amplification tests
qRT-PCR	Quantitative reverse-transcription polymerase chain reaction
CSF	Cerebrospinal fluid
LMIC	Low- and middle-income countries
DETECTR	DNA endonuclease-targeted CRISPR trans reporter
CRISPR	Clustered regularly interspaced short palindromic repeats
Cas	CRISPR-associated
RPA	Recombinase polymerase amplification
RT-RPA	Reverse transcription–recombinase polymerase amplification
PAM	Protospacer-adjacent motif
gRNA	Guide RNA
LFA	Lateral flow assay
LoD	Limit of detection
SARS-CoV-2	Severe acute respiratory syndrome coronavirus 2
HPV	Human papilloma viruses
IAV	Influenza A virus
IBV	Influenza B virus
SFTSV	Severe fever with thrombocytopenia syndrome virus
JEV	Japanese encephalitis virus
SEOV	Seoul virus
DENV	Dengue virus
ZIKV	Zika virus
PFU	Plaque-forming unit
CFU	Colony-forming unit
