# Supplementary material for: A Novel Diagnostic Tool for West Nile Virus Lineage 1a and 2 Using a CRISPR-Cas12a System
Source: Biosensors (Basel). 2025 Dec 10;15(12):807. doi: 10.3390/bios15120807 (PMC12730693; doi:10.3390/bios15120807)
Supplement: Supplementary file 1 [file biosensors-15-00807-s001.zip › biosensors-3976118-supplementary.pdf]

Supplementary Materials

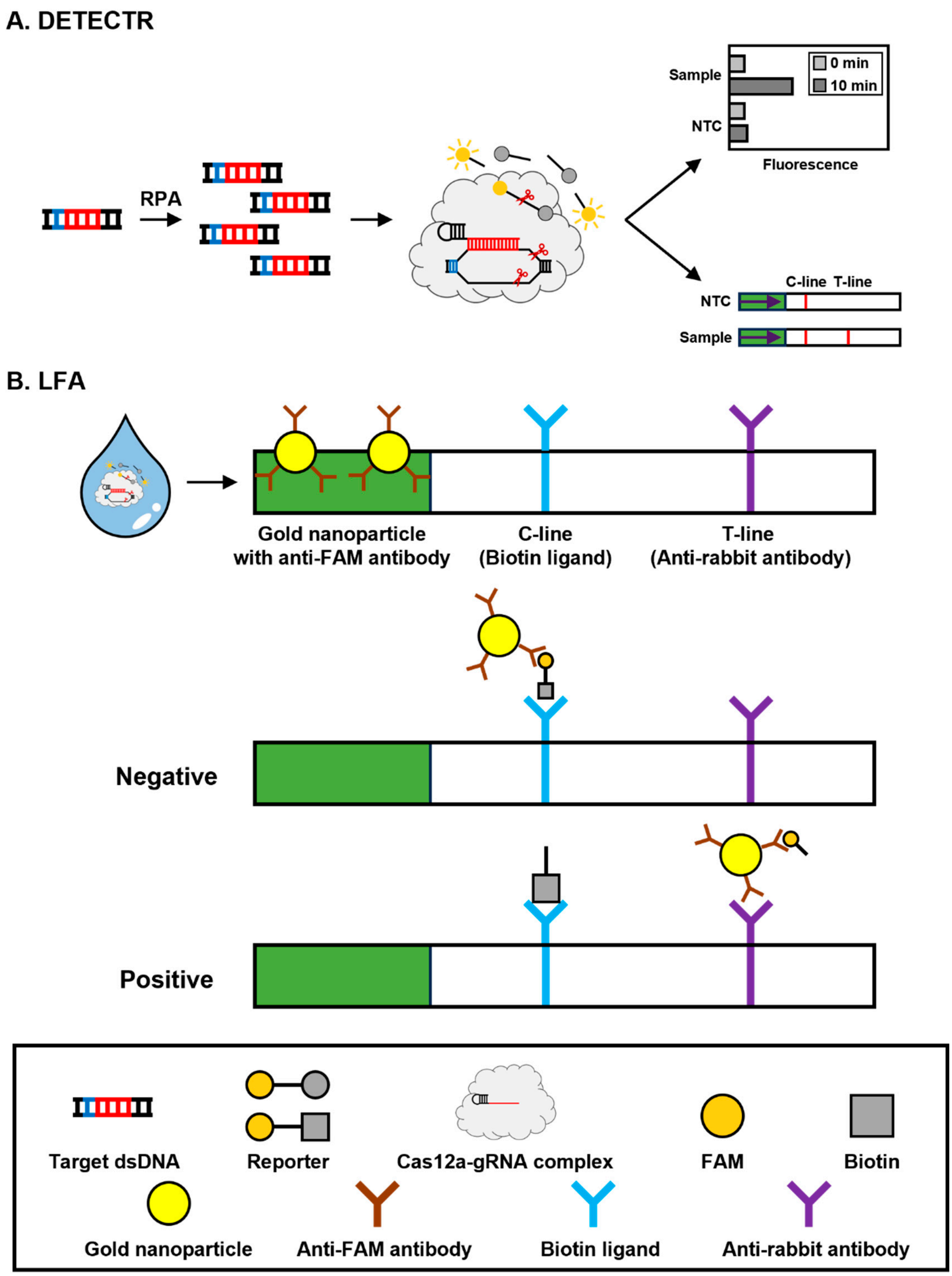

Figure S1. Schematic diagrams of DETECTR and LFA. (**A**) DETECTR; (**B**) Lateral flow assay (LFA).

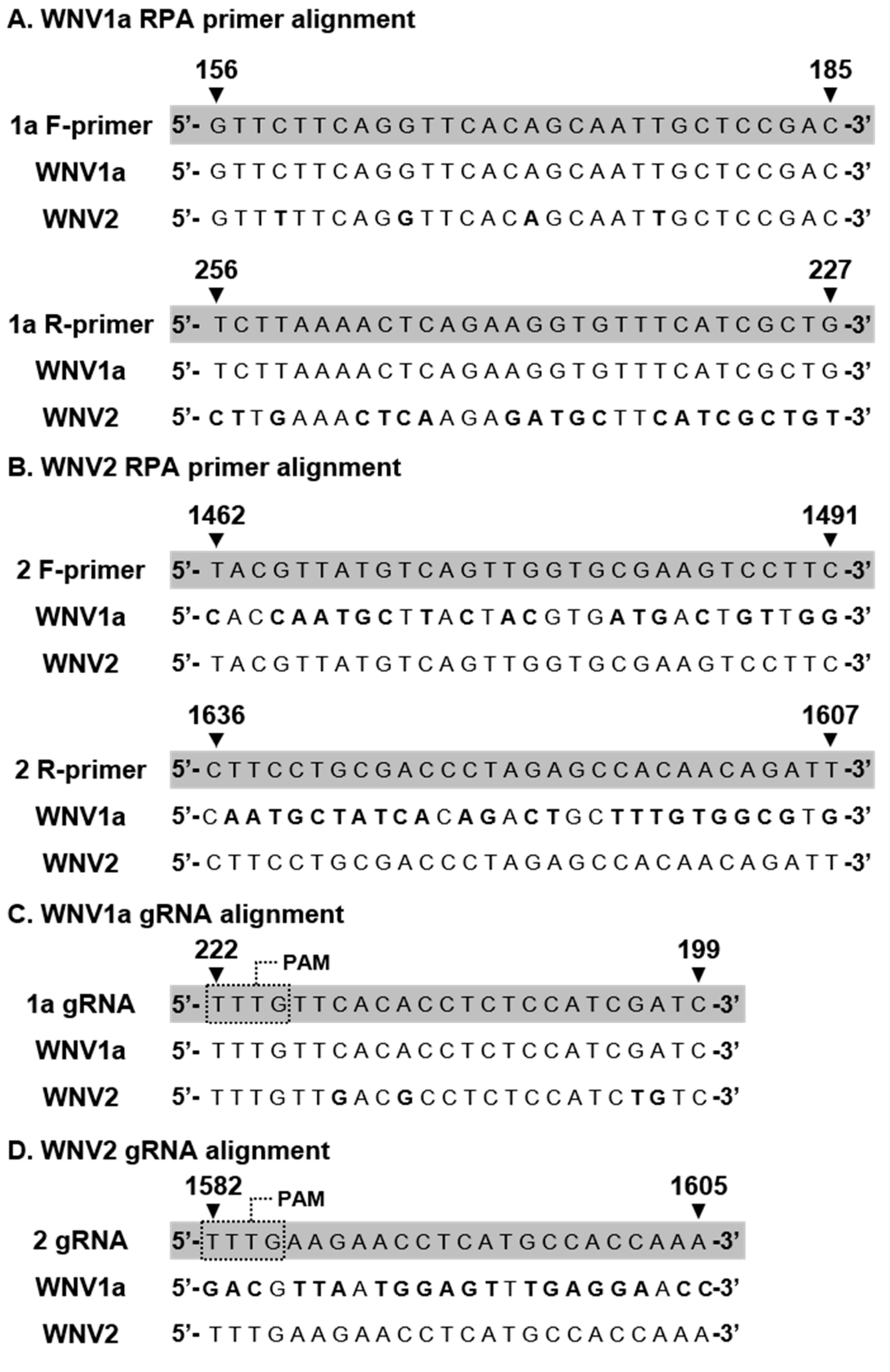

Figure S2. RPA Primer and gRNA alignments for WNV DETECTR. Nucleic acid sequences of (**A** and **B**) RPA primers and (**C** and **D**) gRNAs used in this study are aligned with each other. All RPA reverse primers and WNV1a_gRNA are shown in the reverse-complement orientation.

A. WNV1a alignment

**1a F-primer**	**5'-** G T T C T T C A G G T T C A C A G C A A T T G C T C C G A C **-3'**
	**156** ▼ … **185** ▼
**JF719068**	**5'-** G T T C T T C A G G T T C A C A G C A A T T G C T C C G A C **-3'**
**GU011992**	**5'-** G T T C T T C A G G T T C A C A G C A A T T G C T C C G A C **-3'**
**KF234080**	**5'-** G T T C T T C A G G T T C A C A G C A A T T G C T C C G A C **-3'**
**FJ483549**	**5'-** G T T C T T C A G G T T C A C A G C A A T T G C T C C G A C **-3'**
**FJ483548**	**5'-** G T T C T T C A G G T T C A C A G C A A T T G C T C C G A C **-3'**
**JF719066**	**5'-** G T T C T T C A G G T T C A C A G C A A T T G C T C C G A C **-3'**
**JF719067**	**5'-** G T T C T T C A G G T T C A C A G C A A T T G C T C C G A C **-3'**
**JF719069**	**5'-** G T T C T T C A G G T T C A C A G C A A T T G C T C C G A C **-3'**
**DQ786573**	**5'-** G T T C T T C A G G T T C A C A G C A A T T G C T C C G A C **-3'**
**AY701413**	**5'-** G T T C T T C A G G T T C A C A G C A A T T G C T C C G A C **-3'**
**FJ766331**	**5'-** G T T C T T C A G G T T C A C A G C A A T T G C T C C G A C **-3'**
**FJ766332**	**5'-** G T T C T T C A G G T T C A C A G C A A T T G C T C C G A C **-3'**
**JF707789**	**5'-** G T T C T T C A G G T T C A C A G C A A T T G C T C C G A C **-3'**
**JQ928174**	**5'-** G T T C T T C A G G T T C A C A G C A A T T G C T C C G A C **-3'**
**JX556213**	**5'-** G T T C T T C A G G T T C A C A G C A A T T G C T C C G A C **-3'**
**KF647253**	**5'-** G T T C T T C A G G T T C A C A G C A A T T G C T C C G A C **-3'**
**KC954092**	**5'-** G T T C T T C A G G T T C A C A G C A A T T G C T C C G A C **-3'**
**JN858069**	**5'-** G T T **T** T T C A G G T T C A C A G C A A T T G C T C C G A C **-3'**
**JQ928175**	**5'-** G T T **T** T T C A G G T T C A C A G C A A T T G C T C C G A C **-3'**
**AY701412**	**5'-** G T T C T T C A G G T T C A C A G C A A T T G C T C C G A C **-3'**
**HM152775**	**5'-** G T T C T T C A G G T T C A C A G C A A T T G C T C C G A C **-3'**
**KU588135**	**5'-** **T** T T C T T C A G G T T C A C A G C A A T T G C T C C G A C **-3'**
**KY703854**	**5'-** G T T C T T C A G G T T C A C A G C A A T T G C T C C G A C **-3'**
**JX442279**	**5'-** G T T C T T C A G G T T C A C A G C A A T T G C T C C G A C **-3'**
**JX041634**	**5'-** G T T C T T C A G G T T C A C A G C A A T T G C T C C G A C **-3'**
**AF196835**	**5'-** G T T C T T C A G G T T C A C A G C A A T T G C T C C G A C **-3'**
**GQ851607**	**5'-** G T T C T T C A G G T T C A C A G C A A T T G C T C C G A C **-3'**
**GQ851606**	**5'-** G T T C T T C A G G T T C A C A G C A A T T G C T C C G A C **-3'**
**KT163243**	**5'-** G T T C T T C A G G T T C A C A G C A A T T G C T C C G A C **-3'**
**EU249803**	**5'-** G T T C T T C A G G T T C A C A G C A A T T G C T C C G A C **-3'**
**HM051416**	**5'-** G T T C T T C A G G T T C A C A G C A A T T G C T C C G A C **-3'**
**JX041630**	**5'-** G T T C T T C A G G T T C A C A G C A A T T G C T C C G A C **-3'**
**JX041629**	**5'-** G T T C T T C A G G T T C A C A G C A A T T G C T C C G A C **-3'**
**JX041628**	**5'-** G T T C T T C A G G T T C A C A G C A A T T G C T C C G A C **-3'**
**KC601756**	**5'-** G T T C T T C A G G T T C A C A G C A A T T G C T C C G A C **-3'**
**GQ379161**	**5'-** G T T C T T C A G G T T C A C A G C A A T T G C T C C G A C **-3'**

**1a R-primer**	**5’-** T C T T A A A A C T C A G A A G G T G T T T C A T C G C T G **-3’**
	**256** ▼ … **227** ▼
**JF719068**	**5’-** T C T T A A A A C T C A G A A G G T G T T T C A T C G C T G **-3’**
**GU011992**	**5’-** T C T T A A A A C T C A G A A G G T G T T T C A T C G C T G **-3’**
**KF234080**	**5’-** T C T T A A A A C T C A G A A G G T G T T T C A T C G C T G **-3’**
**FJ483549**	**5’-** T C T T A A A A C T C A G A A G G T G T T T C A T C G C T G **-3’**
**FJ483548**	**5’-** T C T T A A A A C T C A G A A G G T G T T T C A T C G C T G **-3’**
**JF719066**	**5’-** T C T T A A A A C T C A G A A G G T G T T T C A T C G C T G **-3’**
**JF719067**	**5’-** T C T T A A A A C T C A G A A G G T G T T T C A T C G C T G **-3’**
**JF719069**	**5’-** T C T T A A A A C T C A G A A G G T G T T T C A T C G C T G **-3’**
**DQ786573**	**5’-** T C T T A A A A C T C A G A A G G T G T T T C A T C G C T G **-3’**
**AY701413**	**5’-** T C T T A A A A C T C A G A A G G T G T T T C A T C G C T G **-3’**
**FJ766331**	**5’-** T C T T A A A A C T C A G A A G G T G T T T C A T C G C T G **-3’**
**FJ766332**	**5’-** T C T T A A A A C T C A G A A G G T G T T T C A T C G C T G **-3’**
**JF707789**	**5’-** T C T T A A A A C T C A G A A G G T G T T T C A T C G C T G **-3’**
**JQ928174**	**5’-** T C T T A A A A C T C A G A A G G T G T T T C A T C G C T G **-3’**
**JX556213**	**5’-** T C T T A A A A C T C A G A A G G T G T T T C A T C G C T G **-3’**
**KF647253**	**5’-** T C T T A A A A C T C A G A A G G T G T T T C A T C G C T G **-3’**
**KC954092**	**5’-** T C T T A A A A C T C A G A A G G T G T T T C A T C G C T G **-3’**
**JN858069**	**5’-** T C T T A A A A C T C A G A A G G T G T T T C A T C G C T G **-3’**
**JQ928175**	**5’-** T C T T A A A A C T C A **A** A A G G T G T T T C A T C G C T G **-3’**
**AY701412**	**5’-** T C T T A A A A C T C A G A A G G T G T T T C A T C G C T G **-3’**
**HM152775**	**5’-** T C T T A A A A C T C A G A A G G T G T T T C A T **T** G C T G **-3’**
**KU588135**	**5’-** T C T T A A A A C T C A G A A G G T G T T T C A T C G C T G **-3’**
**KY703854**	**5’-** T C T T A A A A C T C A G A A G G T G T T T C A T C G C T G **-3’**
**JX442279**	**5’-** T C T T A A A A C T C A **A** A A G G T G T T T C A T C G C T G **-3’**
**JX041634**	**5’-** T C T T A A A A C T C A **A** A A G G T G T T T C A T C G C T G **-3’**
**AF196835**	**5’-** T C T T A A A A C T C A G A A G G T G T T T C A T C G C T G **-3’**
**GQ851607**	**5’-** T C T T A A A A C T C A **A** A A G G T G T T T C A T C G C T G **-3’**
**GQ851606**	**5’-** T C T T **G** A A A C T C A **A** A A G G T G T T T C A T C G C T G **-3’**
**KT163243**	**5’-** T C T T A A A A C T C A G A A G G T G T T T C A T C G C T G **-3’**
**EU249803**	**5’-** T C T T A A A A C T C A G A A G G T G T T T C A T C G C T G **-3’**
**HM051416**	**5’-** T C T T A A A A C T C A G A A G G T G T T T C A T C G C T G **-3’**
**JX041630**	**5’-** T C T T A A A A C T C A G A A G G T G T T T C A T C G C T G **-3’**
**JX041629**	**5’-** T C T T A A A A C T C A G A A G G T G T T T C A T C G C T G **-3’**
**JX041628**	**5’-** T C T T A A A A C T C A G A A G G T G T T T C A T C G C T G **-3’**
**KC601756**	**5’-** T C T T A A A A C T C A **A** A A G G T G T T T C A T C G C T G **-3’**
**GQ379161**	**5’-** T C T T A A A A C T C A G A A G G T G T T T C A T C G C T G **-3’**

B. WNV2 alignment

Sequence	Start	End	Alignment
**2 F-primer**			5'- TACGTTATGTCAGTTGGTGCGAAGTCCTTC -3'
**KP780839**	1429	1458	5'- TACGTTATGTCAGTTGGTGC**A**AAGTCCTTC -3'
**AY532655**	1462	1491	5'- TACGTTATGTCAGTTGGTGCGAAGTCCTTC -3'
**GQ903680**	1472	1501	5'- **ATGTCATGTCAGT**T**G**G**TGCTA**A**GTCTT**TTC -3'
**KM203863**	1473	1502	5'- TACGTTATGTCAGTTGGTGCGAAGTCCTTC -3'
**JN858070**	1474	1503	5'- TACGTTATGTCAGTTGGTGCGAAGTCCTTC -3'
**KT207792**			5'- TACGTTATGTCAGTTGGTGCGAAGTCCTTC -3'
**KF647251**			5'- TACGTTATGTCAGTTGGTGCGAAGTCCTTC -3'
**KP780838**			5'- TACGTTATGTCAGTTGGTGCGAAGTCCTTC -3'
**KP780837**			5'- TACGTTATGTCAGTTGGTGCGAAGTCCTTC -3'
**KM203862**			5'- TACGTTATGTCAGTTGGTGCGAAGTCCTTC -3'
**KP780840**			5'- TACGTTATGTCAGTTGGTGCGAAGTCCTTC -3'
**KP109692**			5'- TACGTTATGTCAGTTGGTGCGAAGTCCTTC -3'
**KF179640**			5'- TACGTTATGTCAGTTGGTGCGAAGTCCTTC -3'
**KC407673**			5'- TACGTTATGTCAGTTGGTGCGAAGTCCTTC -3'
**KJ883346**			5'- TACGTTATGTCAGTTGGTGCGAAGTCCTTC -3'
**HQ537483**			5'- TACGTTATGTCAGTTGGTGCGAAGTCCTTC -3'
**KF179639**			5'- TACGTTATGTCAGTTGGTGCGAAGTCCTTC -3'
**KC496015**			5'- TACGTTATGTCAGTTGGTGCGAAGTCCTTC -3'
**KT359349**			5'- TACGTTATGTCAGTTGGTGCGAAGTCCTTC -3'
**KC496016**			5'- TACGTTATGTCAGTTGGTGCGAAGTCCTTC -3'
**EF429197**			5'- TACGTTATGTCAGTTGGTGCGAAGTCCTTC -3'
**EF429198**			5'- TACGTTATGTCAGTTGGTGC**T**AAGTCCTTC -3'
**JN393308**			5'- TACGTTATGTCAGTTGGTGCGAAGTCCTTC -3'
**EF429199**			5'- TACGTTATGTCAGTTGGTGC**A**AAGTCCTTC -3'
**KM052152**			5'- TACGTTATGTCAGTTGGTGCGAAGTCCTTC -3'
**EF429200**			5'- TACGTTATGTCAGTTGGTGCGAAGTCCTTC -3'
**KT207791**			5'- TACGTTATGTCAGTTGG**C**GCGAAGTCCTTC -3'
**KJ934710**			5'- TACGTTATGTCAGTTGG**C**GCGAAGTCCTTC -3'
**FJ425721**			5'- TACGTTATGTCAGTTGG**C**GCGAAGTCCTTC -3'
**HM147824**			5'- TACGTTATGTCAGTTGGTGCGAAGTCCTTC -3'
**HM147823**			5'- TA**T**GT**C**ATGTCAGTTGG**G**GCGAAGTCCTTC -3'
**KM203861**			5'- TACGTTATGTCAGTTGGTGCGAAGTCCTTC -3'
**HM147822**			5'- TA**T**GT**C**ATGTC**G**GT**C**GG**C**GCGAAGTCCTT**T** -3'
**DQ176636**			5'- TACGT**A**ATGTCAGT**A**GG**G**GC**A**AAGTCCTTC -3'

2 R-primer 5'-CTTCCTGCGACCCTAGAGCCACAACAGATT-3'

1603 ▼ … 1574 ▼

KP780839 5'-CTTCCTGCGACCCTAGAGCCACAACAGATT-3'

1636 ▼ … 1607 ▼

AY532655 5'-CTTCCTGCGACCCTAGAGCCACAACAGATT-3'

1645 ▼ … 1616 ▼

GQ903680 5'-CTTC**T**TGCGA**T**CC**C**A**AG**GC**T**ACAACAGA**C**T-3'

1647 ▼ … 1618 ▼

KM203863 5'-CTTCCTGCGACCCTAGAGCCACAACAGATT-3'

1648 ▼ … 1619 ▼

JN858070 5'-CTTCCTGCGACCCTA**A**AGCCACAACAGATT-3'
KT207792 5'-CTTCCTGCGACCCTAGAGCCACAACAGATT-3'
KF647251 5'-CTTCCTGCGACCCTAGAGCCACAACAGATT-3'
KP780838 5'-CTTCCTGCGACCCTAGAGCCACAACAGATT-3'
KP780837 5'-CTTCCTGCGACCCTAGAGCCACAACAGATT-3'
KM203862 5'-CTTCCTGCGACCC**C**AGAGCCACAACAGATT-3'
KP780840 5'-CTTCCTGCGACCCTAGAGCCACAACAGATT-3'
KP109692 5'-CTTCCTGCGACCCTAGAGCCACAACAGATT-3'
KF179640 5'-CTTCCTGCGACCCTAGAGCCACAACAGATT-3'
KC407673 5'-CTTCCTGCGACCCTAGAGCCACAACAGATT-3'
KJ883346 5'-CTTCCTGCGACCCTAGAGCCACAACAGATT-3'
HQ537483 5'-CTTCCTGCGACCCTAGAGCCACAACAGATT-3'
KF179639 5'-CTTCCTGCGACCCTAGAGCCACAACAGATT-3'
KC496015 5'-CTTCCTGCGACCCTAGAGCCACAACAGATT-3'
KT359349 5'-CTTCCTGCGACCCTAGAGCCACAACAGATT-3'
KC496016 5'-CTTCCTGCGACCCTAGAGCCACAACAGATT-3'
EF429197 5'-CTTCCTGCGACCCTAGAGCCACAACAGATT-3'
EF429198 5'-CTTCCTGCGACCCTAGAGCCACAACAGATT-3'
JN393308 5'-CTTCCTGCGACCCTAGAGCCACAACAGATT-3'
EF429199 5'-CTTCCTGCGACCCTAGAGCCACAACAGATT-3'
KM052152 5'-CTTCCTGCGACCCTAGAGCCACAACAGATT-3'
EF429200 5'-CTTCCTGCGACCCTAGAGCCACAACAGA**C**T-3'
KT207791 5'-CTTCCTGCGACCCTAGAGCCACAACAGATT-3'
KJ934710 5'-CTTCCTGCGACCCTAGAGCCACAACAGATT-3'
FJ425721 5'-CTTCCTGCGACCCTAGAGCCACAACAGATT-3'
HM147824 5'-CTTCCTGCGACCCTAGAGCCACAACAGATT-3'
HM147823 5'-CTTCCTGCGACCC**C**AGAGCCAC**G**ACAGATT-3'
KM203861 5'-CTTCCTGCGACCCTAGAGCCACAACAGATT-3'
HM147822 5'-CTTC**T**TGCGA**T**CCTA**AG**GC**T**AC**G**ACAGA**C**T-3'
DQ176636 5'-CTTCCTGCGA**T**CC**A**AG**G**GCCAC**T**ACAGATT-3'

Figure S3. Sequence alignments of WNV based on RPA Primer. Nucleic acid sequences of RPA primers used in this study are aligned with strains of each WNV lineage. RPA reverse primers are shown in the reverse-complement orientation. (**A**) WNV1a alignment; (**B**) WNV2 alignment.

A. WNV1a alignment

PAM

1a gRNA	5'- T T T G T T C A C A C C T C T C C A T C G A T C -3'

222 ▼ … 199 ▼

JF719068	5'- T T T G T T C A C A C C T C T C C A T C G A T C -3'
GU011992	5'- T T T G T T C A C A C C T C T C C A T C G A T C -3'
KF234080	5'- T T T G T T C A C A C C T C T C C A T C G A T C -3'
FJ483549	5'- T T T G T T C A C A C C T C T C C A T C G A T C -3'
FJ483548	5'- T T T G T T C A C A C C T C T C C A T C G A T C -3'
JF719066	5'- T T T G T T C A C A C C T C T C C A T C G A T C -3'
JF719067	5'- T T T G T T C A C A C C T C T C C A T C G A T C -3'
JF719069	5'- T T T G T T C A C A C C T C T C C A T C G A T C -3'
DQ786573	5'- T T T G T T C A C A C C T C T C C A T C G A T C -3'
AY701413	5'- T T T G T T C A C A C C T C T C C A T C G A T C -3'
FJ766331	5'- T T T G T T C A C A C C T C T C C A T C G A T C -3'
FJ766332	5'- T T T G T T C A C A C C T C T C C A T C G A T C -3'
JF707789	5'- T T T G T T C A C A C C T C T C C A T C G A T C -3'
JQ928174	5'- T T T G T T C A C A C C T C T C C A T C G A T C -3'
JX556213	5'- T T T G T T C A C A C C T C T C C A T C G A T C -3'
KF647253	5'- T T T G T T C A C A C C T C T C C A T C G A T **T** -3'
KC954092	5'- T T T G T T C A C A C C T C T C C A T C G A T C -3'
JN858069	5'- T T T G T T C A C A C C T C T C C A T C G A T C -3'
JQ928175	5'- T T T G T T C A C A C C T C T C C A T C G A T C -3'
AY701412	5'- T T T G T T C A C A C C T C T C C A T C G A T C -3'
HM152775	5'- T T T G T T C A C A C C T C T C C A T C G A T C -3'
KU588135	5'- T T T G T T C A C A C C T C T C C A T C G A T C -3'
KY703854	5'- T T T G T T C A C A C C T C T C C A T C G A T C -3'
JX442279	5'- T T T G T T C A C A C C T C T C C A T C G A T C -3'
JX041634	5'- T T T G T T C A C A C C T C T C C A T C G A T C -3'
AF196835	5'- T T T G T T C A C A C C T C T C C A T C G A T C -3'
GQ851607	5'- T T T G T T C A C A C C T C T C C A T C G A T C -3'
GQ851606	5'- T T T G T T C A C A C C T C T C C A T C G A T C -3'
KT163243	5'- T T T G T T C A C A C C T C T C C A T C G A T C -3'
EU249803	5'- T T T G T T C A C A C C T C T C C A T C G A T C -3'
HM051416	5'- T T T G T T C A C A C C T C T C C A T C G A T C -3'
JX041630	5'- T T T G T T C A C A C C T C T C C A T C G A T C -3'
JX041629	5'- T T T G T T C A C A C C T C T C C A T C G A T C -3'
JX041628	5'- T T T G T T C A C A C C T C T C C A T C G A T C -3'

226 ▼ … 203 ▼

KC601756	5'- T T T G T T **T A T T** C A C A C C T C T C C A T C -3'
GQ379161	5'- T T T G T T **T A T T** C A C A C C T C T C C A **C** C -3'

B. WNV2 alignment

Sequence	Positions	Sequence (5'→3')
2 gRNA (PAM: TTTG)		5'-TTTGAAGAACCTCATGCCACCAAA-3'
KP780839	1549–1572	5'-TTTGAAGAACCTCATGCCACCAAA-3'
AY532655	1582–1605	5'-TTTGAAGAACCTCATGCCACCAAA-3'
GQ903680	1591–1614	5'-TTTGAAGAACCTCATGCCAC**T**AAA-3'
KM203863	1593–1616	5'-TTTGAAGAACCTCATGCCACCAAA-3'
JN858070	1594–1617	5'-TTTGAAGAACCTCATGCCACCAAA-3'
KT207792		5'-TTTGAAGAACCTCATGCCACCAAA-3'
KF647251		5'-TTTGAAGAACCTCATGCCACCAAA-3'
KP780838		5'-TTTGAAGAACCTCATGCCACCAAA-3'
KP780837		5'-TTTGAAGAACCTCATGCCACCAAA-3'
KM203862		5'-TTTGAAGAACCTCATGCCACCAAA-3'
KP780840		5'-TTTGAAGAACCTCA**C**GCCACCAAA-3'
KP109692		5'-TTTGAAGAACCTCATGCCACCAAA-3'
KF179640		5'-TTTGAAGAACCTCATGCCACCAAA-3'
KC407673		5'-TTTGAAGAACCTCATGCCACCAAA-3'
KJ883346		5'-TTTGAAGAACCTCATGCCACCAAA-3'
HQ537483		5'-TTTGAAGAACCTCATGCCACCAAA-3'
KF179639		5'-TTTGAAGAACCTCATGCCACCAAA-3'
KC496015		5'-TTTGAAGAACCTCATGCCACCAAA-3'
KT359349		5'-TTTGAAGAACCTCATGCCACCAAA-3'
KC496016		5'-TTTGAAGAACCTCATGCCACCAAA-3'
EF429197		5'-TTTGAAGAACCTCATGCCACCAAA-3'
EF429198		5'-TTTGAAGAACCTCATGCCACCAAA-3'
JN393308		5'-TTTGAAGAACCTCATGCCACCAAA-3'
EF429199		5'-TTTGAAGAACCTCATGCCACCAAA-3'
KM052152		5'-TTTGAAGA**G**CC**C**CATGCCACCAAA-3'
EF429200		5'-TTTGAAGAACCTCATGCCACCAAA-3'
KT207791		5'-TTTGAAGAACC**C**CATGCCACCAAA-3'
KJ934710		5'-TTTGAAGAACC**C**CATGCCACCAAA-3'
FJ425721		5'-TTTGAAGAACC**C**CATGCCACCAAA-3'
HM147824		5'-TTTGAAGAACCTCATGCCACCAAA-3'
HM147823		5'-TTTGAAGAACCTCATGCCACCAAA-3'
KM203861		5'-TTTGAAGAACCTCATGCCACCAAA-3'
HM147822		5'-TTTGAAGAACCTCATGC**T**ACCAAA-3'
DQ176636		5'-TTTGAAGA**G**CCTCATGC**T**ACCAAA-3'

Figure S4. Sequence alignments of WNV based on gRNA. Nucleic acid sequences of gRNAs used in this study are aligned with strains of each WNV lineage. WNV1a_gRNA is shown in the reverse-complement orientation. (**A**) WNV1a alignment; (**B**) WNV2 alignment.

Table S1. Primers for *in vitro* transcription

Lineage	Primer	Sequence
WNV1a	T7_F	TAATACGACTCACTATAGGGAGAGGAGGGCCCGGCA
	T7_R	ACTGCTCCTACGCTGGCGATCAGGCCAATCATGACT
WNV2	T7_F	TAATACGACTCACTATAGGGAGATACGTTATGTCAGTTGGTGCGAAGTCCTTC
	T7_R	CAACGCACCTTCCTGCGACCCTAGAGCCACAACAGATT
